# Author Guide for Addressing Animal Methods Bias in Publishing

**DOI:** 10.1002/advs.202303226

**Published:** 2023-08-30

**Authors:** Catharine E. Krebs, Celean Camp, Helder Constantino, Lilas Courtot, Owen Kavanagh, Janine McCarthy, Melanie‐Jasmin Ort, Shaarika Sarasija, Emily R. Trunnell

**Affiliations:** ^1^ Physicians Committee for Responsible Medicine 5100 Wisconsin Ave., NW, Suite 400 Washington DC 20016‐4131 USA; ^2^ Fund for the Replacement of Animals in Medical Experiments Cawley House, 149–155 Canal Street Nottingham NG1 7HR UK; ^3^ Humane Society International Europe Avenue des Arts 50 Brussels 1000 Belgium; ^4^ Animal Free Research UK 27 Old Gloucester Street London WC1N 3AX UK; ^5^ York St. John University Lord Mayor's Walk York YO31 7EX UK; ^6^ Institute for Chemistry and Biochemistry Freie Universität Berlin Arnimallee 20 14195 Berlin Germany; ^7^ BIH Center for Regenerative Therapies Berlin Institute of Health at Charité Universitätsmedizin Berlin Campus Virchow‐Klinikum, Augustenburger Platz 1 13353 Berlin Germany; ^8^ Humane Society International Canada 372 St. Catherine St. West Suite 319 Montreal QC H3B 1A2 Canada; ^9^ People for the Ethical Treatment of Animals 501 Front St. Norfolk VA 23510 USA

**Keywords:** alternatives to animal testing, animal methods bias, peer review, publishing

## Abstract

There is growing recognition that animal methods bias, a preference for animal‐based methods where they are not necessary or where nonanimal‐based methods may already be suitable, can impact the likelihood or timeliness of a manuscript being accepted for publication. Following April 2022 workshop about animal methods bias in scientific publishing, a coalition of scientists and advocates formed a Coalition to Illuminate and Address Animal Methods Bias (COLAAB). The COLAAB has developed this guide to be used by authors who use nonanimal methods to avoid and respond to animal methods bias from manuscript reviewers. It contains information that researchers may use during 1) study design, including how to find and select appropriate nonanimal methods and preregister a research plan, 2) manuscript preparation and submission, including tips for discussing methods and choosing journals and reviewers that may be more receptive to nonanimal methods, and 3) the peer review process, providing suggested language and literature to aid authors in responding to biased reviews. The author's guide for addressing animal methods bias in publishing is a living resource also available online at animalmethodsbias.org, which aims to help ensure fair dissemination of research that uses nonanimal methods and prevent unnecessary experiments on animals.

## Graphical abstract

This guide is created by the Coalition to Illuminate and Address Animal Methods Bias to help authors avoid the negative impacts of reviewers’ preference for animal‐based methods. Intended for researchers who use nonanimal methods, including in vitro models and primary human tissue, it contains resources and information regarding study design, preregistration, manuscript preparation and submission, and reviewer communications.

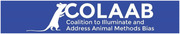

## 1 Introduction

Within scientific publishing, peer review is intended to serve as a mechanism by which manuscripts can be assessed and improved to assure the rigor of published research. However, the system also introduces biases that can affect the likelihood of a manuscript's publication, result in the undertaking of additional experiments, or alter the authors’ communication of their findings to readers.^[^ ^1^ ^]^ We recently defined a type of publishing bias called animal methods bias, which describes a preference for animal‐based methods where they may not be necessary or where nonanimal‐based methods may already be suitable, and which impacts the likelihood and timeliness of a manuscript being accepted for publication. Preliminary evidence of this bias indicates that some researchers perform animal‐based experiments solely in anticipation of reviewer requests for them.^[^ ^2^ ^]^ It has also been shown that reviewers sometimes ask authors to add animal experimental data to studies that otherwise had no animal‐based component, a request that authors often feel is scientifically or ethically unjustified.^[^ ^2^ ^]^ The source of this bias may be attributed to the research enterprises’ reliance on animals and/or a lack of awareness or trust in appropriate innovative nonanimal methods, both of which may result in reviewers’ preferences for animal‐based methods and thus incentivize the use of animals where they are not necessary.

Researchers may choose to use nonanimal experimental models for a variety of reasons, including 1) their ability to reliably mimic human biology and clinical responses, 2) their potential advantages over comparable animal‐based approaches, such as microenvironmental control, longitudinal monitoring, high‐throughput capacity, and patient‐specificity, and 3) their lower resource and ethical burdens.^[^ ^3^, ^4^ ^]^ Reviewers who request validation of findings in an animal model are often basing this request on the presumption that animals are the gold standard in biomedical research, failing to consider that human data or human‐specific models like organ chips may be better suited.^[^ ^5^ ^]^ Furthermore, a reviewer's prescription of a particular method may be inappropriate; while it is often fair to request more evidence, limiting how that evidence is to be gathered can cause unnecessary pressure or stifle innovation. Animal methods bias not only has ethical, time, and cost implications, but it may also contribute to the poor translatability of findings from animal experiments to human clinical outcomes. It can also have career consequences for researchers who use nonanimal methods, causing delays in publication, forcing authors to publish in lower‐impact journals, or leading early‐stage researchers to pursue animal methods because of the impression that they must do so in order to publish and progress their careers. It is therefore important for researchers who use nonanimal methods to understand how to avoid reviewer animal methods bias and address it if necessary.

Following an April 2022 workshop that gathered stakeholders from publishing, academia, industry, government, and non‐governmental organizations to discuss animal methods bias in scientific publishing, a coalition of scientists and advocates formed the Coalition to Illuminate and Address Animal Methods Bias (COLAAB) to build evidence and develop mitigation strategies.^[^ ^6^ ^]^ Members of the COLAAB created this guide to help authors avoid and overcome animal methods bias at various stages throughout the manuscript preparation and submission process. It is intended for life sciences and biomedical researchers who use nonanimal, human‐specific, in silico, or in vitro methods. It contains the following measures, resources, and tools that authors can use before and during manuscript submission, as well as after receiving potentially biased peer reviews:

- A comprehensive bibliography of pertinent resources and references that authors can use at any stage of research;
- Recommendations for authors to design their studies using human‐based models and datasets as validation, to preregister their research plans, and to properly frame and report their findings to prevent reviewers from providing biased reviews;
- Recommendations for determining the best journal for submission and for suggesting suitable reviewers; and
- A step‐by‐step guide for responding to biased reviews in a variety of different scenarios that authors may find themselves, including when reviewers make unsubstantiated critiques or requests for animal experimental validation of findings.

This guide is also available online at animalmethodsbias.org as a living resource that will be updated regularly by members of the COLAAB. While this guide is primarily intended for authors, editors and reviewers may also find it useful.

## 2 Pre‐Submission Preventive Measures

Because animal methods bias can result in publication delays or even manuscript rejection, authors who use nonanimal modeling systems should take preventive steps to avoid these negative outcomes. The following resources and recommendations aim to help authors avert animal methods bias prior to submission, from the study design stage onwards. A comprehensive bibliography of resources presented as a shared Zotero library may be useful for authors during any stage of research, from study design to manuscript preparation to responding to reviewers. See Section 2.1 for access to this resource.

### 2.1 Bibliography of Relevant Resources

This bibliography consists of a variety of resources, including:

- Animal research policy and legislation across several governing regions;
- Literature about preparing manuscripts, publishing biases, and peer review;
- Scholarship about animal model validity and translatability; and
- Reviews, methods papers, and examples of successful applications of nonanimal modeling systems.

Any author can access this library, and any researcher or advocate can contribute to the library by applying for contributing access.

The bibliography, which is formatted as a shared Zotero library, can be accessed here: https://www.zotero.org/groups/4803770/animal_method_bias_mitigation_group/library.

### 2.2 Using Human‐Based Models and Datasets

The first step to counter animal methods bias in publishing is to anticipate its potential to occur. When preparing a manuscript for a nonanimal‐based study, authors should discuss the inadequacies of animal experimentation and the availability of suitable nonanimal method alternatives in the relevant field of research. Authors should also consider performing additional nonanimal experiments to corroborate findings. These measures can proactively counter downstream reviewer bias.

**Table** **1** provides potential resources that may be useful for authors when designing studies and preparing manuscripts to help avert animal methods bias during peer review. It includes nonanimal method search strategies, Biorepositories and datasets, and information on collaborating with clinicians, published by governmental, industrial, and academic sources around the world.

**Table 1:** Human‐based models, datasets, and other resources.

Resource	Weblink
Search for animal alternatives and 3Rs databases (with the country of origin in parentheses)	
The National Agricultural Library's Animal Welfare Information Center (AWIC; USA)	https://www.nal.usda.gov/services/literature‐searching‐animal‐use‐alternatives
Resource guide for helping members of Georgetown University comply with IACUC requirements and perform searches based on the 3Rs (USA)	https://guides.dml.georgetown.edu/alternatives/strategies
University of North Carolina Chapel Hill 3R keyword searches (USA)	https://guides.lib.unc.edu/animal‐alternatives/3R‐keywords
University of Hawai'i at Mānoa IACUC Resources and Searching Tips (USA)	https://hslib.jabsom.hawaii.edu/c.php?g=576949&p=4000892
Norecopa: 3R Guide database	https://norecopa.no/databases‐guidelines/3r‐guide‐database
University of Pennsylvania bibliographic database: indexes the literature and specialty databases on 3R topics (USA)	https://guides.library.upenn.edu/3rs/databases
Animals in Science 3R's Database: compiled by Research Animal Training (UK)	https://resources.researchanimaltraining.com/articles/categories/3r‐database
USDA National Agriculture Library guidance on building and conducting a 3R's alternatives literature search (USA)	https://www.nal.usda.gov/services/literature‐searching‐animal‐use‐alternatives
The European 3R's Society guide to nonanimal method compliant associations and centers in Europe and internationally	https://eusaat.eu/the‐3rs‐society/3rs‐associations‐centers/3rs‐europe/
Biorepositories and datasets	
The Central Biorepository at the University of Michigan Medical School Office of Research (USA)	https://research.medicine.umich.edu/our‐units/central‐biorepository
California Institute for Regenerative Medicine Induced Pluripotent Stem Cell Repository (USA)	https://www.cirm.ca.gov/researchers/ipsc‐repository
Alzheimer's Disease Neuroimaging Initiative (ADNI): MRI and PET images, genetics, cognitive tests, CSF, and blood biomarkers to define the progression of Alzheimer's disease (USA)	https://adni.loni.usc.edu/
Adolescent Brain Cognitive Development (ABCD) Study: the largest long‐term study of brain development and child health in the USA	https://abcdstudy.org/
The All of Us Research Program: health data from a diverse group of participants from across the USA	https://www.researchallofus.org/
The NIH NeuroBioBank: human post‐mortem brain tissue (USA)	https://neurobiobank.nih.gov/
The Genotype‐Tissue Expression (GTEx) project: a resource database of genotype and tissue‐specific gene expression levels in human tissues (USA)	https://www.gtexportal.org/home/
The Human Cell Atlas (HCA): human cell profile database (USA)	https://www.humancellatlas.org/data‐coordination‐2/
The (SUM) Breast Cancer Cell Line Knowledge Base (SLKBase): the SUM human breast cancer cell lines and over 50 other human breast cancer cell lines	https://sumlineknowledgebase.com/
The National Centralized Repository for Alzheimer's Disease and Related Dementias (NCRAD): supports research focused on the etiology, early detection, and therapeutic development of Alzheimer's disease and related dementias (USA)	https://ncrad.iu.edu/
Allen Human Brain Atlas: A unique multimodal atlas of the human brain, integrating anatomic and genomic information including microarray data, in situ hybridization image data, and MRI data (USA)	https://human.brain‐map.org/
Chan Zuckerberg CELL by GENE (CZ CELLxGENE) Discover: reference‐quality data to understand the functionality of human tissues at the cellular level with (USA)	https://cellxgene.cziscience.com/
The European Union Reference Laboratory for Alternatives to Animal Testing	https://data.jrc.ec.europa.eu/collection/id‐0088
European Bank for induced pluripotent Stem Cells (EBiSC): a centralized, not‐for‐profit iPSC bank providing researchers across academia and industry with access to scalable, cost‐efficient, and consistent, high‐quality iPSC lines and derived products for new medicines development	https://ebisc.org/
Toxicology and pharmacology resources	
TOXNET	https://www.nlm.nih.gov/toxnet/index.html
Downloadable Computational Toxicology Data from the Environment Protection Agency (USA)	https://www.epa.gov/chemical‐research/downloadable‐computational‐toxicology‐data
Lexi‐Comp Online: clinical pharmacology databases compiled by Dalhousie University (Canada)	https://dal.ca.libguides.com/c.php?g=257016&p=4942508
A comprehensive list of in silico and in vitro testing resources provided by PETA Science Consortium International (Germany)	https://www.thepsci.eu/links‐resources/
Clinical research and collaboration	
NIH Policy and Guidelines on The Inclusion of Women and Minorities as Subjects in Clinical Research	https://grants.nih.gov/policy/inclusion/women‐and‐minorities/guidelines.htm
Twelve Lessons Learned for Effective Research Partnerships Between Patients, Caregivers, Clinicians, Academic Researchers, and Other Stakeholders	https://www.thepsci.eu/links‐resources/
Bridging the Translational Research Gap: A Successful Partnership Involving a Physician and a Basic Scientist	https://europepmc.org/article/PMC/3519100

Disclaimer: These resources are not managed by the authors of this guide, and their inclusion in this table is not an official endorsement.

In addition, Table S1 (Supporting Information) provides a list of companies offering platforms, tools, and other products for researchers to conduct studies and experimental validation with nonanimal methods. Authors can refine searches by filtering for companies offering specific kinds of methods or models or for those which are available in their country. This list is non‐exhaustive, and the COLAAB aims to continually update its online form at animalmethodsbias.org. Researchers are encouraged to contact the COLAAB with any additional companies or amendments. Another list of companies offering nonanimal method products can be found on this In Silico/In Vitro Testing Resources webpage.^[^ ^7^ ^]^

### 2.3 Preregistering a Research Plan

Preregistration is the process of specifying a research plan before conducting the study and submitting it to a registry or as a registered report to be peer‐reviewed and published in a journal.^[^ ^8^ ^]^ It can improve research quality, transparency, and reproducibility, potentially increasing the likelihood of a final manuscript's acceptance.^[^ ^9^ ^]^ Preregistration enables researchers to demonstrate their goals and justify their methods before data is collected or analyzed and allows early input from peers.^[^ ^10^ ^]^ It is already mandatory for clinical trials.

Because nonanimal methods are relatively novel compared to animal‐based approaches, confidence in their ability to model human biology and clinical characteristics is still being established within the scientific community.^[^ ^11^ ^]^ Preregistration's effect on research quality, transparency, and reproducibility makes it particularly beneficial in the context of nonanimal methods as trust continues to build in these novel approaches. Furthermore, because it strengthens the justification for a study's design prospectively, preregistration can bolster a nonanimal‐based study against unjustified reviewer critiques about the methods used. It may even prevent peer reviewers from requesting animal experiments to validate findings as it establishes a complete research plan to which authors can refer. Preregistration allows a more complete scientific record of a study, whereby sharing and publishing negative results is encouraged. Historically, negative results have been disfavored by the publishing system and missing in published scientific literature.^[^ ^12^ ^]^ Reporting negative results is of immense value during subsequent studies when authors must justify experiments and make the case for not performing or repeating animal experiments for further validation. See Section 2.3.1 for resources about preregistration, including registries and journals that offer registered report article types. For a list of journals that welcome negative results, see **Table** **2**.

**Table 2:** Journals that publish studies with negative results.

Journal	Publisher	Scope	Link
PLOS ONE: Missing Pieces Collection	PLOS	PLOS ONE considers all work that makes a contribution to the field, independent of impact. This includes negative findings which are valuable to the community in cases where the result is illuminating in the context of previous work.	https://everyone.plos.org/2015/02/25/positively‐negative‐new‐plos‐one‐collection‐focusing‐negative‐null‐inconclusive‐results/
ACS Omega	ACS Publications	ACS Omega is an open‐access global publication for scientific articles that describe new findings in chemistry and interfacing areas of science, without any perceived evaluation of immediate impact.	https://pubs.acs.org/journal/acsodf
F1000Research	F1000	F1000Research publishes articles and other research outputs reporting basic scientific, scholarly, translational, and clinical research across the physical and life sciences, engineering, medicine, social sciences, and humanities. F1000Research is a scholarly publication platform set up for the scientific, scholarly, and medical research community.	https://f1000research.com/
PeerJ	PeerJ	The peer‐reviewed & Open Access journal publishing primary research and reviews in biology, life sciences, environmental sciences, and medicine.	https://peerj.com/
BMC Research Notes	BioMed Central	BMC Research Notes is an open‐access journal publishing peer‐reviewed contributions from across all scientific and clinical disciplines, including intriguing initial observations, updates to previous work and established methods, valid negative results, and scientific data sets and descriptions. BMC Research Notes does not make editorial decisions on the basis of the interest of a study or its likely impact.	https://bmcresnotes.biomedcentral.com/

#### 2.3.1 Preregistration Resources

Center for Open Science information about preregistration: https://www.cos.io/initiatives/prereg

How to submit preregistration to the Open Science Framework registry: https://help.osf.io/article/158‐create‐a‐preregistration

Center for Open Science information about registered reports: https://www.cos.io/initiatives/registered‐reports

Journals that offer the registered report article type: the Center for Open Science Registered Reports database (https://www.cos.io/initiatives/registered‐reports)

### 2.4 Preparing a Manuscript that Describes Nonanimal Methods

Careful study design and diligent reporting can help avoid a significant amount of pushback from reviewers who prefer animal methods. It is crucial to provide detailed protocols and methods in a manuscript so that proper evaluation of the work can be performed. Word limits and journal formatting may hinder such writing, but there are tools and best practices to help optimize manuscript preparation. The advice in Section 2.4.1 can help authors prepare manuscripts and may increase the chance of acceptance at a target journal. This advice has been created based on comments from reviewers and editors on how to avoid requests for additional data prior to publication.

#### 2.4.1 Advice for Authors in Preparing Nonanimal Manuscripts

##### Model Justification

The authors should explain why their experimental model of choice is suitable for the research question. In particular, the human biological relevance of the methods should be made clear. If the research area is dominated by in vivo animal methods, an exact and detailed explanation should be provided to outline why animals are not suitable for the given hypothesis and subsequent work.

##### Validation

Robustness of the evidence presented is important for advancing a field forward and for reviewers to evaluate a study's merit. The most persuasive evidence of a study's robustness is the use of different or complementary methods that corroborate findings. In research fields where animals are routinely used, requests from peer reviewers to validate findings with animal data may be anticipated. To preempt such requests, authors may include a nonanimal experimental validation step in their original study design (see Section 2.2: Using Human‐Based Models and Datasets). If such a validation step was not included, authors may bolster the validity of their findings in other ways, such as by corroborating their findings with other peer‐reviewed research. These studies could be previous animal studies, but studies with human subjects or clinical data would provide even more powerful evidence and maximize the translatability of findings.

##### Reproducibility

Authors must ensure that other researchers can replicate their study procedures and findings by being fully transparent and detailed with methodological procedures and data. This is especially important for studies that employ nonanimal methods as scientific confidence in these approaches is still being built.^[^ ^11^ ^]^ All details of the study design need to be provided. Guidelines for reporting on in vitro studies have been described elsewhere and provide valuable information for authors to improve the reproducibility and reliability of their results (for example^[^ ^13^, ^14^, ^15^ ^]^). Details such as the number of replicates, blinding procedures, temperatures, times, reagents used, and many more are crucial for authors to include. Adherence to these guidelines may not be a requirement for a particular study, but their implementation can enhance a study's quality and make the results more reliable and credible to the broader scientific community and reviewers.

##### Drawing Conclusion

Overselling findings and overstating facts can lead to flawed reproducibility and false claims. Conclusions must be drawn accurately from the data and evidence provided. New and exciting methods can tempt researchers to overstate the power of the method used and may draw criticism or even rejection from editors and reviewers. Overstating could also lead to requests for further validation experiments, especially validation in animals. Along the same lines, it is also important for authors to adequately address the limitations of their study, which can be framed as areas of future investigation.

## 3 Manuscript Submission

At the editorial and peer review stages, when animal methods bias manifests, it may result in a lengthy revision period or ultimately rejection if authors fail to comply with requests for animal experiments. To minimize these undesirable outcomes, the COLAAB has compiled the following resources for use during manuscript submission, including a list of journals with a track record of publishing nonanimal studies for consideration upon submission, as well as recommendations for suggesting reviewers.

### 3.1 Submitting the Manuscript to an Appropriate Journal

Submitting to an appropriate journal may not guarantee a total avoidance of the effects of animal methods bias, but it can reduce them. Choosing the right journal is important for other reasons, like maximizing the study's reach and impact. To determine if a journal is the right fit, it is important to check the study fits within its scope, and recent issues should also be checked for articles that contain a similar methodology as the study ready for submission. Considering the journal's audience is also crucial to this decision.

Other compelling journal characteristics may also be considered, such as open access, open peer review, and how likely the journal is to accept negative results (Table 2). Open peer review can refer to various aspects of the peer review process, including 1) open identities when authors and reviewers are aware of each other's identities; 2) open reports, when review reports are published with the final manuscript; 3) open participation, when the wider community can contribute to the review; or 4) open pre‐review manuscripts, when manuscripts are made immediately available (for example, through preprint servers like bioRxiv^[^ ^16^ ^]^) ahead of journal peer review.^[^ ^17^ ^]^ Among other benefits, open peer review may improve the quality, transparency, and reproducibility of research, and it may prevent or mitigate biases, including animal methods bias.^[^ ^18^ ^]^ Improving academic publishing and the peer review process is a hotly contested issue, though.^[^ ^19^ ^]^ The effect of open review on animal methods bias has not yet been investigated or demonstrated, and it may introduce other biases, such as racial, gender, or seniority biases.^[^ ^20^ ^]^ These are all important factors when considering where to submit.

The journals listed in **Table** **3** each have a stated scope that is explicitly inclusive of nonanimal studies or receptive to them. This list is not exhaustive and it is also available as a living resource online at animalmethodsbias.org where it will be updated periodically.

**Table 3:** A non‐exhaustive list of journals receptive to nonanimal methods.

Journal	Publisher	Scope	Link
ACS Biomaterials & Science Engineering	American Chemical Society	Modeling and informatics tools for biomaterials; synthesis and modulation of new biomaterials; bioinspired and biomimetic approaches to biomaterials; biomaterial interfaces and interactions; health risk studies of biomaterials; manufacturing, technology, and tissues in the context of biomaterials; bioresponsive biomaterials, bioelectronics, and bioMEMS; biomaterials‐based devices and prosthetics; regenerative medicine; genetic designs and bioengineering	https://pubs.acs.org/journal/abseba
ACS Nano	American Chemical Society	ACS Nano publishes comprehensive articles on synthesis, assembly, characterization, theory, and simulation of nanostructures (nanomaterials and assemblies, nanodevices, and self‐assembled structures), nanobiotechnology, nanofabrication, methods and tools for nanoscience and nanotechnology, and self‐ and directed‐assembly.	https://pubs.acs.org/journal/ancac3
Advanced Materials	Wiley	Advanced Materials publishes the latest progress in materials at the cutting edge of the chemistry and physics of functional materials.	https://onlinelibrary.wiley.com/journal/15214095
Advanced Science	Wiley	Advanced Science is an interdisciplinary premium open‐access journal covering fundamental and applied research in materials science, physics, chemistry, medical and life sciences, as well as engineering.	https://onlinelibrary.wiley.com/journal/21983844
ALTEX	Springer	ALTEX—Alternatives to Animal Experimentation publishes academic articles on the development and implementation of alternatives to the use of animals for scientific purposes and informs on international developments in this field.	https://www.altex.org/index.php/altex
Applied In Vitro Toxicology	Mary Ann Liebert, Inc.	Applied In Vitro Toxicology provides the latest research on the development, validation, and application of new and innovative in vitro testing methods for predicting adverse effects of drugs, chemicals, and products in the pharmaceutical, chemical, or personal care industries	https://home.liebertpub.com/publications/applied‐in‐vitro‐toxicology/626
ATLA	SAGE journals	ATLA – Alternatives to Laboratory Animals‐ intends to cover all aspects of the development, validation, implementation, and use of alternatives to laboratory animals in biomedical research and toxicity testing.	https://journals.sagepub.com/home/ATL
Bioinformatics	Oxford Academic	Bioinformatics focuses on new developments in genome bioinformatics and computational biology.	https://academic.oup.com/bioinformatics
Bioinspiration & Biomimetics	IOP Publishing	Bioinspiration & Biomimetics publishes research involving the study and distillation of principles and functions found in biological systems that have been developed through evolution, and application of this knowledge to produce novel and exciting basic technologies and new approaches to solving scientific problems.	https://iopscience.iop.org/journal/1748‐3190
Experimental Biology and Medicine	SAGE Journals	Experimental Biology and Medicine is dedicated to the publication of multidisciplinary and interdisciplinary research in the biomedical sciences. Articles represent cutting‐edge research at the overlapping junctions of the biological, physical, and engineering sciences that impact the health and welfare of the world's population.	https://journals.sagepub.com/home/ebm
Frontiers in Bioengineering and Biotechnology	Frontiers	Frontiers in Bioengineering and Biotechnology is a forum for research involved in the process of bridging the gap between discovery in the basic sciences and its clinical application.	https://www.frontiersin.org/journals/bioengineering‐and‐biotechnology
Frontiers in Computational Neuroscience	Frontiers	Frontiers in Computational Neuroscience promotes theoretical modeling of brain function and fosters multidisciplinary interactions between theoretical and experimental neuroscience.	https://www.frontiersin.org/journals/computational‐neuroscience
In Vitro Toxicology	Frontiers	The In Vitro Toxicology specialty section publishes original research and review papers on any topic pertinent to the dynamic field of in vitro toxicology.	https://www.frontiersin.org/journals/toxicology/sections/in‐vitro‐toxicology
Journal of Translational Medicine	Springer Nature	Journal of Translational Medicine publishes articles focusing on information derived from human experimentation to optimize the communication between basic and clinical science.	https://translational‐medicine.biomedcentral.com/
Lab on a Chip	Royal Society of Chemistry	Lab on a Chip publishes work related to miniaturization, at the micro‐ and nano‐scale, of interest to a multidisciplinary readership. The journal publishes work at the interface between physical technological advancements and high‐impact applications.	https://www.rsc.org/journals‐books‐databases/about‐journals/lab‐on‐a‐chip/
Microfluidics and Nanofluidics	Springer Nature	Microfluidics and Nanofluidics explore all aspects of microfluidics, nanofluidics, and lab‐on‐a‐chip science and technology. The journal seeks to improve the fundamental understanding of microfluidic and nanofluidic processes, examining the current state of research and development and the latest applications.	https://www.springer.com/journal/10404
Nature Communications	Nature	Nature Communications is an open‐access, multidisciplinary journal dedicated to publishing high‐quality research in all areas of the biological, health, physical, chemical, and Earth sciences.	https://www.nature.com/ncomms/
Nature Nanotechnology	Nature	Nature Nanotechnology covers research into the design, characterization, and production of structures, devices, and systems that involve the manipulation and control of materials and phenomena at atomic, molecular, and macromolecular scales.	https://www.nature.com/nnano/
Organs‐on‐a‐Chip	Science Direct	Organs‐on‐a‐Chip publishes research and development in the field of organs, tissues, and organoids on chips and their application.	https://www.sciencedirect.com/journal/organs‐on‐a‐chip
Scientific Reports	Nature	Scientific Reports is an open‐access journal publishing original research from across all areas of the natural sciences, psychology, medicine, and engineering.	https://www.nature.com/srep/
Science Translational Medicine	Science	Science Translational Medicine promotes human health by providing a forum for communicating the latest research advances from biomedical, translational, and clinical researchers from all established and emerging disciplines relevant to medicine.	https://www.science.org/journal/stm
Stem Cell Reports	Cell Press	Stem Cell Reports focuses on original research with conceptual or practical advances that are of broad interest to stem cell biologists and clinicians.	https://www.cell.com/stem‐cell‐reports/home
Toxicology in Vitro	Science Direct	Toxicology in Vitro focuses on the application and use of in vitro and *in silico* Systems for toxicological evaluations (collectively described as New Approach Methodologies (NAMs)).	https://www.sciencedirect.com/journal/toxicology‐in‐vitro

There are useful tools that can also help to find a good potential match for a manuscript. **Table** **4** lists journal selectors that can help determine an appropriate set of journals for a manuscript based on its abstract. SciRev is another valuable web resource where authors share their experiences with specific journals’ review processes and can provide insight as to whether a particular journal may be a good fit for a given manuscript.^[^ ^21^ ^]^

**Table 4:** Journal selectors.

Journal selector	Link
Jane: Journal/Author Name Estimator	https://jane.biosemantics.org/
Journal Guide	https://www.journalguide.com/
End Note Manuscript Matcher	https://endnote.com/product‐details/manuscript‐matcher/
Elsevier Journal Finder	https://journalfinder.elsevier.com/
Springer Nature Journal Suggester	https://journalsuggester.springer.com/
Wiley Journal Finder	https://journalfinder.wiley.com/search?type=match

### 3.2 Suggesting Suitable Reviewers

Authors are often provided an opportunity to suggest reviewers for their manuscript. Authors should take advantage of the opportunity to suggest reviewers because 1) finding peer reviewers is increasingly difficult for editors; 2) having appropriate reviewers with the expertise and capacity to objectively evaluate a study is beneficial to authors; and 3) avoiding animal‐biased reviewers can save time, energy, and resources.

Some authors might fear that an editor will purposefully *not* use reviewers they suggest; however, this may be unfounded. While there is no guarantee that editors will use the suggested reviewers, there is little evidence demonstrating that editors actively avoid them. If given the opportunity to provide suggested reviewers in the cover letter, authors are encouraged to explain in one or two sentences why they were chosen. In Section 3.2.1, a list of recommendations for suggesting reviewers is provided. Some journals also provide the opportunity to suggest reviewers not to use. This can be a valuable opportunity to let editors know of researchers that may provide negative, biased, or outright hostile reviews.

#### 3.2.1 Recommendations for Finding Reviewers to Suggest

##### 3.2.1.1 Adapted from “How to Find Reviewers.”^[^ ^22^ ^]^

1. Try to suggest 3–6 reviewers to provide to editors when you submit your manuscript.
2. The most important characteristic of a suggested reviewer is appropriate expertise. Look for experts in the same field or methodology. A good place to start is to look for authors in your manuscript's reference list. You may not find researchers who have expertise in *every* area of your study; it is fine to suggest reviewers with expertise in a *part* of the study, like the experimental system or the tissue or disease type.
3. Look for experts who understand the value of nonanimal methods. You may wish to look specifically for researchers who do not use animals, but it may be more important to look for researchers who have been explicit about the benefits of nonanimal methods and the limitations of animal‐based methods. Check the introductions and methods of their recent papers for this sort of evidence.
4. Look for demographic, global, and career‐stage diversity. Diversity improves the quality and impact of science.^[^ ^23^ ^]^ The inclusion of diverse peer reviewers is important for addressing structural racism, sexism, and other forms of oppression in biomedical research and academia^[^ ^24^ ^]^ and may improve the quality of reviews. Early‐career reviewers may be more likely to say yes to review, and they may be more likely to provide a high‐quality review.^[^ ^25^ ^]^ An effective way to find early‐career reviewers is to look within the lab or among the collaborators of a more prominent, senior researcher.
5. Do not suggest reviewers at your institution or who you have worked with in the last two years. This would present a serious conflict of interest.
6. Do not contact the reviewers; that is the editor's job.

## 4 Responding to Biased Reviews

Every peer review is different and there is no one‐size‐fits‐all response to reviews. Nonetheless, the following guidelines can help authors to identify biased reviews and address them confidently.

**Figure 1:**
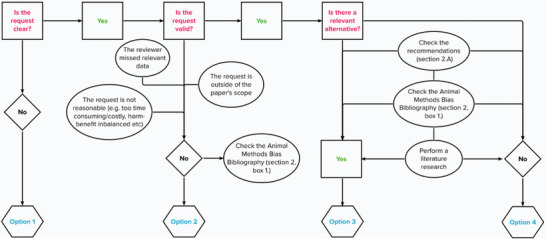
Flowchart for authors to determine how to respond to biased reviews.

A reviewer's animal methods bias can manifest in a variety of ways, including 1) the use of biased language (Section 4.1); 2) recommendations to add references to animal‐based research or amend critiques on the limitations of animal methods; and 3) requests for authors to perform animal‐based experiments, often as a way to validate the results presented in the manuscript. Authors should not feel obliged to comply with such reviews, especially in the case of reviewer requests to perform animal experiments. Rather, authors should thank reviewers for their time and respond to all comments using clear, persuasive, and precise language.^[^ ^26^ ^]^ More detailed scenarios and guidelines for responding are provided in **Figure** **1** and Supporting Information.

1 **The Power of Language**

Language that seems to be objective, free from prejudice, or stating plain facts may still perpetuate bias and harm against non‐human animals used in research. For example, the term “animal model” could be seen as reductionist language portraying sentient beings as products from a biomedical catalog. The following phrase, “a cancer mouse model was produced using CRISPR/Cas9,” conveys a scientific methodology in a straightforward way, but it hides the fact that the mice were genetically altered in utero and born to develop severe tumors that cause them pain, immobility, and lack of appetite and playfulness. Being aware of language's hidden harms can begin to shift the narrative from laboratory animals as mere inanimate resources to one that recognizes their sentience and moral status.^[^ ^27^ ^]^ In addition to perpetuating biases, peer reviewers’ language can harm researchers. Peer reviews can sometimes contain condescending or excessively opinionated language.^[^ ^28^ ^]^ Reviews containing insulting comments or *ad hominem* attacks are unprofessional and authors should report these to journal editors.^[^ ^29^ ^]^

To adequately respond to reviewers, authors must assess reviews for clarity, relevance, and feasibility. The flowchart in Figure 1 summarizes successive steps that demonstrate how to navigate various scenarios involving a reviewer's request for additional animal experiments. We have provided suggested language that authors can use in their responses in Supporting Information.

First, authors should ensure that the purpose of a reviewer's request is well understood. If the review is unclear, authors should ask for clarification and further explanation (see Option 1 in Figure 1 and Supporting Information).

Second, authors should analyze the relevance and feasibility of the request carefully. Here are some questions to ask:

- Is the request within the scope of the article or the goal of the experiment?
- Does it pertain to data already in the article that have been missed or misinterpreted?
- Would fulfilling the request be excessively burdensome in terms of time, resources, or cost?
- Is the reviewer requesting validation experiments that have already been cited or performed using a different method?

In such situations, authors may respond by clarifying the scope of their research and identifying the evidence already in the article, amending the article as necessary if anything is unclear, or explaining why the reviewer's request is not otherwise justified (see Option 2 in Figure 1 and Supporting Information).

It may also be the case that the reviewer makes a relevant suggestion—that an additional experiment *would* improve the study—but the authors feel that the use of animals in this context would not provide significant benefits to the study. This may be because the suggested experiment is not related to a major finding or because the suggested experiment has demonstrated low translational value in the field of study. In such cases, authors can argue that the balance of harm versus benefits, which is enshrined in the legislations of many countries as a prerequisite to use animals in research, would not be met and point to the relevant legislation or regulation (see Option 3 in Figure 1 and Supporting Information and the Zotero library (Section 2.1.) for references to legislation and other resources relevant to this section).

Authors may determine that the reviewer has identified real shortcomings in the article or the experiments that need to be addressed. In this case, authors should seek to respond to these points while avoiding the use of animals. If feasible, this may be done by conducting additional experiments that would address the reviewer(s) concerns without using animals and instead using resources such as human‐based models and datasets (see Option 3 in Figure 1 and Supporting Information and Section 3.1. for more information on nonanimal experimental resources).

Alternatively, authors may agree with the shortcomings identified by the reviewer, but there is no alternative to the animal experiment suggested. In this case, authors can explain how the suggested experiment would be valuable but that due to the lack of an appropriate model, it is infeasible (see Option 4 in Figure 1 and Supporting Information).

## 5 Conclusion and Future Directions

This resource aims to help authors prevent and address animal methods bias during manuscript peer review. It lays out nonanimal, human‐based models and datasets that authors can use to validate their findings without using animals. It conveys the importance of preregistration in preventing biased reviews and provides resources for authors to preregister their studies. It provides guidance for properly framing manuscripts, finding the right journal for submission, and suggesting reviewers. Finally, it provides step‐by‐step guidance for authors to respond to biased reviews, complete with suggested response language and a bibliography of relevant resources, including animal welfare legislation in different countries, literature on the limitations of animal research, and literature on nonanimal, human‐specific experimental systems. While these resources are intended primarily for authors to address animal methods bias in publishing, they may also be useful for facing animal methods bias in other contexts, such as applying for grants or preparing doctoral dissertations.

As previously mentioned, ensuring the reproducibility of nonanimal methods, especially newly developed techniques, can enhance their reliability and credibility within the broader scientific community. Therefore, we encourage authors to consider publishing detailed protocols within the original manuscript (i.e., in the supplementary material), in a repository such as figshare,^[^ ^30^ ^]^ or with a protocol journal in the post‐acceptance period, to increase the visibility of the original study and to improve validation and adoption of the new nonanimal methods. Similarly, the open sharing of data is also important for study transparency and reproducibility and allows other researchers to build on findings. Some journals require authors to submit datasets to appropriate data repositories; regardless, we encourage authors to do so (see, for example, this Data Repository Guidance for more information^[^ ^31^ ^]^).

Bias mitigation is not straightforward. Nevertheless, there has been no previous set of recommendations for authors to mitigate the negative effects of animal methods bias, and the tools provided herein serve as an important starting point. This guidance document is also presented as a living resource online at animalmethodsbias.org where it will be updated periodically as relevant information evolves. The COLAAB welcomes any suggestions to add to, remove from, or amend this guidance. We encourage researchers to contact the corresponding author with such suggestions. The COLAAB also intends to explore ways of building a community around animal methods bias, particularly for authors to share experiences or reviewer communications and provide peer‐to‐peer support.

As much as animal methods bias can be prevented and addressed on the part of the authors, it is a systemic issue that will require systemic solutions. These include actions on the part of publishers to implement guidelines and bias training for editors and reviewers and on the part of funding agencies to prioritize research and infrastructure in nonanimal approaches.^[^ ^6^ ^]^ While this guide may be useful for editors and reviewers, interventions that are more tailored to their responsibilities will likely be more effective. The COLAAB aims to continue to explore these and other mitigation strategies.

## Conflict of Interest

The authors declare no conflict of interest.

## Supporting information

Supporting Information

## References

[1] S. Haffar , F. Bazerbachi , M. H. Murad , Mayo Clin. Proc. 2019, 94, 670. 30797567 10.1016/j.mayocp.2018.09.004

[2] C. E. Krebs , A. Lam , J. McCarthy , H. Constantino , K. Sullivan , ALTEX 2023, 10.14573/altex.2210212 37463512

[3] D. E. Ingber , Nat. Rev. Genet. 2022, 23, 467. 35338360 10.1038/s41576-022-00466-9 PMC8951665

[4] A. Loewa , J. J. Feng , S. Hedtrich , Nat. Rev. Bioeng. 2023, 1, 545. 10.1038/s44222-023-00063-3 PMC10173243 37359774

[5] D. E. Ingber , Adv. Sci. 2020, 7, 2002030. 10.1002/advs.202002030 PMC7675190 33240763

[6] C. E. Krebs , C. Camp , H. Constantino , L. Courtot , O. Kavanagh , S. B. Leite , J. Madden , A. Paini , B. Poojary , I. J. Tripodi , E. R. Trunnell , ALTEX 2022. 10.14573/altex.2210211 36317507

[7] In Silico /In Vitro Testing Resources, https://www.thepsci.eu/links‐resources/, (accessed: May 2023).

[8] B. A. Nosek , C. R. Ebersole , A. C. DeHaven , D. T. Mellor , Proc. Natl. Acad. Sci. U. S. A. 2018, 115, 2600. 29531091 10.1073/pnas.1708274114 PMC5856500

[9] PLOS P, https://plos.org/open‐science/preregistration/, (accessed: January 2023).

[10] American Psychological Association, Preregistration, https://www.apa.org/pubs/journals/resources/preregistration, (accessed: January 2023).

[11] A. J. van der Zalm , J. Barroso , P. Browne , W. Casey , J. Gordon , T. R. Henry , N. C. Kleinstreuer , A. B. Lowit , M. Perron , A. J. Clippinger , Arch. Toxicol. 2022, 96, 2865. 35987941 10.1007/s00204-022-03365-4 PMC9525335

[12] D. Mehta , Nature. https://www.nature.com/articles/d41586‐019‐02960‐3

[13] D. Pamies , M. Leist , S. Coecke , G. Bowe , D. G. Allen , G. Gstraunthaler , A. Bal‐Price , F. Pistollato , R. B. M. de Vries , H. T. Hogberg , T. Hartung , G. Stacey G , ALTEX Altern. Anim. Exp. 2022, 39, 30. 10.14573/altex.2111011 34882777

[14] Co‐operation, and Development, Guidance Document on Good In Vitro Method Practices, https://www.oecd‐ilibrary.org/environment/guidance‐document‐on‐good‐in‐vitro‐method‐practices‐givimp_9789264304796‐en, (accessed: February 2023).

[15] Z. Zhao , X. Chen , A. M. Dowbaj , A. Sljukic , K. Bratlie , L. Lin , E. L. S. Fong , G. M. Balachander , Z. Chen , A. Soragni , M. Huch , Y. A. Zeng , Q. Wang , H. Yu , Nat. Rev. Methods Primers 2022, 2, 94. 37325195 10.1038/s43586-022-00174-y PMC10270325

[16] The RIVER working group, Reporting In Vitro Experiments Responsibly – the RIVER Recommendations, MetaArXiv, 10.31222/osf.io/x6aut.

[17] T. Ross‐Hellauer , F1000 Res. 2017, 6, 588. 10.12688/f1000research.11369.1 PMC5437951 28580134

[18] R. Bruce , A. Chauvin , L. Trinquart , P. Ravaud , I. Boutron , B. M. C. Med. 2016, 14, 85. 10.1186/s12916-016-0631-5 PMC4902984 27287500

[19] A. Ahmed , A. Al‐Khatib , Y. Boum , H. Debat , A. Gurmendi Dunkelberg , L. J. Hinchliffe , F. Jarrad , A. Mastroianni , P. Mineault , C. R. Pennington , J. A. Pruszynski , Nat. Hum. Behav 2023, 7, 1021. 37443268 10.1038/s41562-023-01637-2

[20] N. Shoham , A. Pitman , B. J. Psych. Adv. 2021, 27, 247.

[21] SciRev – Review the scientific review process, https://scirev.org/ (accessed: May 2023).

[22] How to find reviewers, https://www.springer.com/gp/authors‐editors/editors/how‐to‐find‐reviewers/32890, (accessed: January 2023).

[23] T. H. Swartz , A.‐G. S. Palermo , S. K. Masur , J. A. Aberg , J. Infect. Dis. 2019, 220, S33. 31430380 10.1093/infdis/jiz174 PMC6701939

[24] H. Else , J. M. Perkel , Nature 2022, 602, 566. 35197624 10.1038/d41586-022-00426-7

[25] ScientistSeesSquirrel, Early career researchers make great peer reviewers. How can we get more of them?, https://scientistseessquirrel.wordpress.com/2017/01/23/early‐career‐researchers‐make‐great‐peer‐reviewers‐how‐can‐we‐get‐more‐of‐them/, (accessed: January 2023).

[26] N. Parletta , How to respond to difficult or negative peer‐reviewer feedback, https://www.nature.com/nature‐index/news‐blog/how‐to‐respond‐difficult‐negative‐peer‐reviewer‐feedback, (accessed: January 2023).

[27] K. Smith , Hum. Biol. 2011, 83, 261. 21615289 10.3378/027.083.0207

[28] N. J. Silbiger , A. D. Stubler , Peer J. 2019, 7, e8247. 31844596 10.7717/peerj.8247 PMC6911688

[29] G. Conroy . Q&A Linda Beaumont: Journals should take action against toxic peer reviews, https://www.nature.com/nature‐index/news‐blog/linda‐beaumont‐research‐journals‐should‐take‐action‐against‐toxic‐peer‐reviews, (accessed: January 2023).

[30] figshare – credit for all your research, https://figshare.com/, (accessed: May 2023).

[31] Scientific Data, Data Repository Guidance, https://www.nature.com/sdata/policies/repositories, (accessed: May 2023).

