# Supplementary material for: Author Guide for Addressing Animal Methods Bias in Publishing
Source: Adv Sci (Weinh). 2023 Aug 30;10(30):2303226. doi: 10.1002/advs.202303226 (PMC10602554; doi:10.1002/advs.202303226)
Supplement: Supplementary file 1 — Supporting Information [file ADVS-10-2303226-s001.pdf]

ADVANCED SCIENCE
Open Access

Supporting Information

for *Adv. Sci.*, DOI 10.1002/advs.202303226

Author Guide for Addressing Animal Methods Bias in Publishing

Catharine E. Krebs, Celean Camp, Helder Constantino, Lilas Courtot, Owen Kavanagh, Janine McCarthy, Melanie-Jasmin Ort, Shaarika Sarasija and Emily R. Trunnell*

Supporting Information

Author Guide for Addressing Animal Methods Bias in Publishing

Catharine E. Krebs, Celean Camp, Helder Constantino, Lilas Courtot, Owen Kavanagh, Janine McCarthy, Melanie-Jasmin Ort, Shaarika Sarasija, and Emily R. Trunnell*

Table S1. Companies offering nonanimal modelling platforms.

**NAME**	**METHOD/MODEL**	**WEBSITE**	**COUNTRY**
4DCell	Organ-On-A-Chip	https://www.4dcell.com	France
Abcalis	Antibodies	https://abcalis.com/	Germany
Absolute Antibody	Antibodies	https://absoluteantibody.com/	USA & Canada
Accio BiobankOnline	Human biosamples	https://biobankonline.com/	UK
Alcyomics	Ex - vivo human skin	https://alcyomics.com/	UK
Altis Biosystems	In Vitro Testing	https://www.altisbiosystems.com	USA
Alvéole	Bio-engineering in vitro	https://www.alveolelab.com/ fr/	France
Alveolix	Organ-On-A-Chip	https://www.alveolix.com	Switzerland
Ananda	Organ-On-A-Chip	https://www.anandadevices.com	Canada
Aptagen	Aptamers	https://www.aptagen.com/	USA
Aracari Biosciences	Organ-On-A-Chip	https://aracaribio.com/	US
AvantGen	Antibodies	https://www.avantgen.com/	USA
AXOL	Stem Cells	https://axolbio.com/	UK and USA

AxoSim	Organ-On-A-Chip	https://axosim.com	USA
Azar Innovations	Organ-On-A-Chip	https://azar-innovations.com/	Netherlands
BeonChip	Organ-On-A-Chip	https://beonchip.com	Spain
BioIVT	In Vitro Testing	https://bioivt.com/	USA
BIOLIFE4D	Tissue Engineering	https://biolife4d.com	USA
Biomillenia	Microfluidics for microbiology	https://biomillenia.com/	France
BiomimX SRL	Organ-On-A-Chip	https://www.biomimx.com	Italy
Bit Bio	Stem Cells	https://bit.bio	UK
BrainXell	Stem Cells	https://brainxell.com/	US
Catapult	Drug Discoveries	https://md.catapult.org.uk/	UK
Cellasys	Organ-On-A-Chip	https://www.cellasys.com	Ireland
Cellink	3D Bioprinting	https://www.cellink.com/	USA, EU, and more
Celvivo	3D In Vitro Models	https://celvivo.com/	Denmark
Certara	Simulator	https://www.certara.com	USA
Cherry Biotech	Organ-On-A-Chip	https://www.cherrybiotech.com	France
Chiron	Organ-On-A-Chip	https://www.chrn.co/	Netherland
Clinic'n'cell	Ex vivo clinical trials	https://clinicncell.com/	France
CN-Bio	Organ-On-A-Chip	https://cn-bio.com	United Kingdom
Cortism	Transcranial alternating current stimulation (tACS)	https://www.corstim.com/	France
CSIRO	Organ-On-A-Chip	https://www.csiro.au/	Australia
Curi Bio	Drug discovery platform	https://www.curibio.com/	Seattle
Cyprotex	Organ-On-A-Chip	https://www.cyprotex.com	USA
Cytena	In Vitro Automation	https://www.cytena.com/	USA & EU

Cytoprotex	In vitro and in silico ADME-Tox	https://www.cyprotex.com/	USA & UK
Discovery Life Sciences	Biospecimen and biomarkers	https://www.dls.com/	Multiple Sites
Draper	Organ-On-A-Chip	https://www.draper.com/	US
Eden MedTech	Organ-On-A-Chip	https://eden-microfluidics.com/	France
Elveflow	Organ-On-A-Chip	https://www.elveflow.com	France
Emulate	Organ-On-A-Chip	https://www.emulatebio.com	USA
Episkin	Tissue Engineering	https://www.episkin.com/	France
Epthelix	In vitro Respiratory Testing	https://www.epithelix.com/	Switzerland
Flocel Inc	Flow-based 3D In Vitro Models	https://www.flocel.com/	USA
Fluigent	Organ-On-A-Chip	https://www.fluigent.com/	France
Geneva Antibody Facility	Antibodies	https://www.unige.ch/medecine/antibodies/	Switzerland
Genoskin	Ex - vivo human skin	https://www.genoskin.com	France
Hemogenix	In Vitro Testing	https://www.hemogenix.com	USA
HemoShear	Drug discovery platform	https://hemoshear.com	USA
Hesperos Inc.	Organ-On-A-Chip	https://hesperosinc.com/	USA
HookeBio	3D In Vitro Models	https://hookebio.com/	Ireland
Humabiologics, Inc	Human-Derived Biomaterial	https://humabiologics.com/	USA
iBiochips	Organ-On-A-Chip	https://ibiochips.com	USA
Iktos	AI and drug development	https://iktos.ai/	UK, USA, and France
iLoF	AI Personalised Medicine	https://ilof.tech/	UK & Portugal

ImmuOne	In Vitro Testing	https://immuone.com/	UK
Insphero	Organ-On-A-Chip	https://insphero.com	USA, Canada and EU
Intravacc	Vaccine Development	https://www.intravacc.nl	Netherlands
Keratify	3D In Vitro Models	https://www.keratify.com/	UK
Kirkstall	Organ-On-A-Chip	https://www.kirkstall.com/	United Kingdom
Kiyatec	3D In Vitro Models	https://www.kiyatec.com/	US
Labskin	Human skin models	https://www.labskin.co.uk/	UK
Lena Biosciences	Organ-On-A-Chip	https://www.lenabio.com/	US
Luxembourg Institute of Science and Technology	3D In Vitro Models	https://www.list.lu	UK
MatTek	Organ-On-A-Chip	https://www.mattek.com/	USA
Mesobiotech	Organ-On-A-Chip	https://mesobiotech.com	France
Metatissue	Human-based products	https://metatissue.com/	Portugal
MicroMatrices	3D In Vitro Models	https://www.micromatrices.com/	UK
Micronit	Organ-On-A-Chip	https://www.micronit.com	Netherlands & Germany
Mimetas	Organ-On-A-Chip	https://www.mimetas.com	Netherlands
Multus	Xeno-free media	https://www.multus.bio/	UK
Ncardia	Stem Cells	https://www.ncardia.com/	Netherlands
Netri	Organ-On-A-Chip	https://netri.com/	France
New Cells Biotech	Organ-On-A-Chip	https://newcellsbiotech.co.uk/	UK

Nortis Bio	Organ-On-A-Chip	https://www.nortisbio.com	USA
Obatala Sciences	Organ-On-A-Chip	https://www.obatalasciences.com/	USA
OMRF Antibody Facility	Antibodies	https://omrf.org/research-faculty/core-facilities/human-antibody-core-facility/	USA
Organovo	3D Bioprinting	https://organovo.com	USA
Poietis	3D Bioprinting	https://poietis.com/	France
Porsolt	In Vitro Testing	https://www.porsolt.com	France
Prellis	Antibodies	https://www.prellisbio.com/	USA
Qkine	Growth Factors for Stem Cells, Organoid etc	https://qkine.com/	UK an USA
React4Life	3D In Vitro Models	https://www.react4life.com/	Italy
Reprocell	Organ-On-A-Chip	https://www.reprocell.com	USA
Revivocell	Organ-On-A-Chip	https://revivocell.com/	UK
Rubynanomed	MPS for Metastasis Detection	https://rubynanomed.com/	Portugal
Scinora	De-Novo Cell Culture	https://scinora.com/	Switzerland
S-Cubed	Semiconductor Technology	https://www.s-cubed-global.com	USA
Simulations Plus	Simulator	https://www.simulations-plus.com	USA
Specifica	Antibodies	https://www.specifica.bio/	USA
STEMCELL Technologies	Human-Relevant Products	https://www.stemcell.com/	USA, EU and more
Stemnovate	Drug Discovery	https://stemnovate.co.uk/	UK
SunBioscience	3D In Vitro Models	https://sunbioscience.ch/	Switzerland
Swift Analytical	3D bioprinting	https://www.swiftanalytical.com/	UK

Synaxys	Neuro-engineering	https://synaxys.com/	France
Synvivo	Organ-On-A-Chip	https://www.synvivobio.com	USA
Taihoya	Dynamic Culture Systems	https://taihoya.com/en	Taiwan
Talk Biosamples	Human Biosamples	https://www.talk-bio.com/	UK
Tara Biosystems	Stem Cell, Tissue Engineering	https://tarabiosystems.com/	USA
Ten Bio	Human Skin Models	https://ten-bio.com/	UK (Scotland)
Tessara Therapeutics	3D Brain Models	https://www.tessaratherapeutics.com/	Australia
Tissuse	Organ-On-A-Chip	https://www.tissuse.com/	Germany
TNC Bio	Xeno-free Cell Culture	https://tncbio.com/	Netherland
Transcell Biologics	Stem Cells	http://transcellbio.science/	India
Tresars	Antibodies	https://tresars.com/	UK
uFluidix Inc.	Organ-On-A-Chip	https://ufluidix.com	Canada
XCellR8	In Vitro Testing	https://x-cellr8.com/	UK

Suggested text that authors may use in their responses to reviewers and editors

Refer to the flowchart in Figure 1 for descriptions of each scenario. Authors should feel free to edit and adapt the example text as they see fit and to personalize each response. Authors should always keep responses professional and courteous. Authors should fill in the [bracketed text] as it appropriately applies to their manuscript and reviews.

Option 1: *The request is unclear.*

Kindly ask the reviewer to provide more explanation.

For example:

A suggestion that we would like to take great care to address is the request that we perform experiments on animals in order to [validate/verify/replicate] our findings. Unfortunately, we find the [reviewer's/reviewers'] request to be unclear [add any specifics as to what is unclear]. Could they please explain further?

Option 2: *You understand the request and do not think you need to perform additional experiments.*

Provide a detailed explanation, with references, for:

- Why the evidence you have provided is reliable and valid
- Why the additional experiments are outside the scope of the article, infeasible, or unethical

For example:

One suggestion that we would like to take great care to address is the request that we perform experiments on animals in order to [validate/verify/replicate] our findings. We do not find this request to be scientifically or ethically justified for the following reasons.

There are legal and regulatory mandates to reduce and replace animal use where possible.

> [For U.S.:] In the U.S., the Animal Welfare Act requires that investigators consider alternatives to procedures that may cause more than momentary or slight pain or distress to the animals (U.S. Congress, 1985) and the U.S. Government Principles for Utilization and Care of Vertebrate Animals Used in Testing, Research, and Training endorses consideration of nonanimal alternatives to reduce or replace the use of animals (National Research Council, 2011).
>
> [For EU:] In the EU, Directive 2010/63/EU states: "wherever possible, a scientifically satisfactory method or testing strategy, not entailing the use of live animals, shall be used instead of a procedure," and: "The use of animals for scientific or educational purposes should therefore only be considered where a non-animal alternative is unavailable" (European Parliament, 2010).
>
> [For UK:] "The Animals (Scientific Procedures) Act 1986 stipulates that "wherever possible, a scientifically satisfactory method or testing strategy not entailing the use of protected animals must be used instead of a regulated procedure" (Parliament of the United Kingdom, 1986).
>
> [For Canada:] According to the Canadian Council on Animal Care, "the use of animals in research, teaching, and testing is acceptable only if it promises to contribute to understanding of fundamental biological principles, or to the development of knowledge that can reasonably be expected to benefit humans or animals" (Canadian Council on Animal Care, 1989).

In the light of [this principle/these principles], [I/we] do not believe that the use of animals would be necessary nor ethical in this instance.

Importantly, experiments on animals would not lend scientific credibility to our findings. Broadly speaking, the translatability of experiments on animals to findings in humans is much lower than was previously thought (Pound and Bracken, 2014; Akhtar, 2015; Frangogiannis, 2022). Fundamental differences in physiology and genetics among species, critical disparities between human disease and the animal models created to study them, inherent effects of inbreeding and life in a laboratory, and the inability of animals to mimic human life histories and comorbidities severely limits the applicability of experiments on animals to the human-relevant questions we sought to answer. Specifically, [add information about poor translatability of animal models in the focus area of the study; check our shared Zotero library (Box 1) for useful references].

Animal methods bias, or a preference for animal-based methods where they may not be necessary or where nonanimal-based methods may be suitable, which affects the likelihood of a manuscript being accepted for publication, is increasingly being recognized as a problem in publishing (Krebs, Camp, et al., 2022; Krebs, Lam, et al., 2023). The consequences of animal methods bias may include the unnecessary use of animals in scientific procedures, delay in publication, delay in the acceptance of nonanimal methods, the proliferation of less-translatable animal models, and "a misattribution of success in biomedical advances due to the use of animal-based experiments, despite the foundational work being done in nonanimal human-specific experimental systems" (Krebs, Camp, et al., 2022; Krebs, Lam, et al., 2023).

For these reasons, we respectfully decline the [reviewer's/reviewers'] request to perform experiments on animals and hope that the editors will agree with this decision.

Option 3: *You understand the request, agree that you need to perform additional experiments, and know a valid alternative to the suggested animal experiment.*

Offer an alternative option to the suggested animal experiment with a nonanimal methodology and describe the plan and the reasons why this approach will provide results that are more relevant.

For example:

A suggestion that we would like to take great care to address is the request that we perform experiments on animals in order to [validate/verify/replicate] our findings. The [reviewer/reviewers] suggested that we conduct *in vivo* experiments in animals for this purpose, but we believe we can achieve results that are more relevant to the research question by using nonanimal methods. Broadly speaking, the translatability of experiments on animals to findings in humans is much lower than was previously thought (Pound and Bracken, 2014; Akhtar, 2015; Frangogiannis, 2022). Fundamental differences in physiology and genetics among species, critical disparities between human disease and the animal models created to study them, inherent effects of inbreeding and life in a laboratory, and the inability of animals to mimic human life

histories and comorbidities severely limits the applicability of experiments on animals to the human-relevant questions we sought to answer. Specifically, [add information about poor translatability of animal models in the focus area of the study; check our shared Zotero library (Box 1) for useful references].

Additionally, there are legal and regulatory mandates to reduce and replace animal use where possible. For these reasons, we plan to add the following [experiment/experiments] instead of performing experiments on animals: [describe the nonanimal experiments you will perform].

Therefore, we accept the reviewer's request to [validate/verify/replicate] our results but will do so by the methods described above.

Option 4: *You understand and agree with the request, but there is no alternative to the animal experiment suggested.*

Explain how the suggested experiment would be valuable but that due to the lack of an appropriate model, it is infeasible.

For example:

A suggestion that we would like to take great care to address is the request that we perform experiments on animals in order to [validate/verify/replicate] our findings. The [reviewer/reviewers] suggested that we conduct *in vivo* experiments in animals for this purpose. Being cautious of the legal and regulatory mandates to reduce and replace animal use where possible, and the increased availability and scientific relevance of alternative methods, we have been thorough in looking for any alternative to the animal experiment suggested by the [reviewer/reviewers]. Unfortunately, we did not find an alternative and thus believe this request to be infeasible.

For this reason, we respectfully decline the [reviewer's/reviewers'] request to perform experiments on animals and hope that the editors will agree with this decision.

References

Akhtar, A. (2015). The Flaws and Human Harms of Animal Experimentation. Camb Q Healthc Ethics 24, 407–419. https://doi.org/10.1017/S0963180115000079.

Canadian Council on Animal Care (1989). Ethics of Animal Investigation. Ottowa: Canadian Council on Animal Care. Available at: https://ccac.ca/Documents/Standards/Policies/Ethics_of_animal_investigation.pdf [Accessed November 29, 2022].

European Parliament (2010). Directive 2010/63/EU. Available at: http://data.europa.eu/eli/dir/2010/63/oj/eng [Accessed October 19, 2022].

Frangogiannis, N. G. (2022). Why animal model studies are lost in translation. J Cardiovasc Aging. https://doi.org/10.20517/jca.2022.10.

Krebs, C. E., Camp, C., Constantino, H. et al. (2022). Proceedings of a workshop to address animal methods bias in scientific publishing. ALTEX. https://doi.org/10.14573/altex.2210211.

Krebs, C. E., Lam, A., McCarthy, J. et al. (2023). A survey to assess animal methods bias in scientific publishing. ALTEX - Alternatives to animal experimentation. https://doi.org/10.14573/altex.2210212.

National Research Council (2011). Guide for the Care and Use of Laboratory Animals. 8th ed. Washington, DC: National Academies Press. Available at: https://grants.nih.gov/grants/olaw/guide-for-the-care-and-use-of-laboratory-animals.pdf.

Parliament of the United Kingdom (1986). Animals (Scientific Procedures) Act 1986. Statute Law Database. Available at: https://www.legislation.gov.uk/ukpga/1986/14/data.pdf [Accessed November 29, 2022].

Pound, P. and Bracken, M. B. (2014). Is animal research sufficiently evidence based to be a cornerstone of biomedical research? BMJ 348, g3387–g3387. https://doi.org/10.1136/bmj.g3387.

U.S. Congress (1985). 7 USC Ch. 54: TRANSPORTATION, SALE, AND HANDLING OF CERTAIN ANIMALS. Available at: https://uscode.house.gov/view.xhtml?path=/prelim@title7/chapter54&edition=prelim [Accessed October 19, 2022].
